# Getting confused: learning reduces parasitoid foraging efficiency in some environments with non-host-infested plants

**DOI:** 10.1007/s00442-019-04384-2

**Published:** 2019-03-30

**Authors:** Ilka Vosteen, Nika van den Meiracker, Erik H. Poelman

**Affiliations:** 1 Laboratory of Entomology, Wageningen University, Wageningen, The Netherlands; 2 Present Address: Department of Chemical Ecology, Bielefeld University, Bielefeld, Germany

**Keywords:** HIPVs, Olfaction, Volatiles, *Cotesia glomerata*, Non-reward learning

## Abstract

Foraging animals face the difficult task to find resources in complex environments that contain conflicting information. The presence of a non-suitable resource that provides attractive cues can be expected to confuse foraging animals and to reduce their foraging efficiency. We used the parasitoid *Cotesia glomerata* to study the effect of non-host-infested plants and associative learning on parasitoid foraging efficiency. Inexperienced *C. glomerata* did not prefer volatiles emitted from host (*Pieris brassicae*)-infested plants over volatiles from non-host (*Mamestra brassicae*)-infested plants and parasitoids that had to pass non-host-infested plants needed eight times longer to reach the host-infested plant compared to parasitoids that had to pass undamaged plants. Contrary to our expectations, oviposition experience on a host-infested leaf decreased foraging efficiency due to more frequent visits of non-host-infested plants. Oviposition experience did not only increase the responsiveness of *C. glomerata* to the host-infested plants, but also the attraction towards herbivore-induced plant volatiles in general. Experience with non-host-infested leaves on the contrary resulted in a reduced attraction towards non-host-infested plants, but did not increase foraging efficiency. Our study shows that HIPVs emitted by non-host-infested plants can confuse foraging parasitoids and reduce their foraging efficiency when non-host-infested plants are abundant. Our results further suggest that the effect of experience on foraging efficiency in the presence of non-host-infested plants depends on the similarity between the rewarding and the non-rewarding cue as well as on the completeness of information that parasitoids have acquired about the rewarding and non-rewarding cues.

## Introduction

Animals that forage for small and inconspicuous food organisms face the difficult task to find these resources in complex environments (Crowder and Cooper 1982; Randlkofer et al. 2010; Wäschke et al. 2013). To handle the enormous complexity of available information and to make adaptive foraging choices, insects need to filter relevant from irrelevant information. They may achieve this by employing search templates which describe innate or learned preferences for stimuli that are likely correlated with the occurrence of the desired resource (Aartsma et al. 2019). Insects with a very narrow search template face the risk to ignore resource-related cues, if these are altered by biotic or abiotic factors. A broad search template on the contrary may result in attraction to non-resource cues, thereby causing the insect to waste time and energy on the inspection and handling of a non-resource, a process we will refer to as confusion.

Herbivore-induced plant volatiles (HIPVs) are important foraging cues for many insectivores, including birds, predatory mites, entomopathogenic nematodes and various predatory insects and parasitoids (Mumm and Dicke 2010; Ali et al. 2011; Amo et al. 2013). Responses towards HIPVs have been intensively studied in parasitoids and nearly every tested species preferred volatiles from herbivore-attacked plants to those from undamaged plants in two-choice assays (Mumm and Dicke 2010; Oudenhove et al. 2017). To use HIPVs as long-range cues under natural conditions, parasitoids have to be able to identify the blend of interest within a complex odour background (Wäschke et al. 2014; Meiners 2016; Aartsma et al. 2017). Most of the HIPV-blend components are produced by a wide range of plant species (Pichersky and Gershenzon 2002), but the composition of the HIPV blend may depend on the plant species and the identity of the attacking herbivore (Dicke and Hilker 2003; Hare 2011; Danner et al. 2018). Ratios of the blend components likely determine if a blend is perceived as attractive (Beyaert and Hilker 2014) and parasitoids may use subtle differences in volatile profiles to distinguish between plants attacked by host or non-host herbivores (McCormick et al. 2012). A meta-analysis shows that highly specialised parasitoids have a narrow search template and mainly respond to specific volatile blends that are induced by their hosts, while more generalist parasitoids, which attack hosts from different taxonomic families, have broader search template and also react to volatile blends induced by the feeding of non-host herbivores or mechanical damage (Oudenhove et al. 2017).

It has been shown that inexperienced parasitoids often do not distinguish between HIPVs emitted by host- and non-host-infested plants when herbivores are of the same feeding guild (Geervliet et al. 1996; Shiojiri et al. 2000; Vos et al. 2001; Bukovinszky et al. 2012; Peñaflor et al. 2017) and may, thus, be confused by non-host-infested plants (Vos et al. 2006; de Rijk et al. 2013). In a field mesocosm experiment, parasitization rate by the parasitoid *Cotesia glomerata* was reduced by 50% in the presence of non-host-infested plants compared to undamaged plants, indicating a decreased foraging efficiency (Bukovinszky et al. 2012). As foraging efficiency is directly related to parasitoid fitness (Thiel and Hoffmeister 2009), response to HIPVs of non-host-infested plants may have severe fitness consequences for parasitoids, depending on the time investment in foraging on non-host-infested plants (Vos et al. 2006). A strong fitness reduction could be expected if parasitoids land on the non-host-infested plant to examine feeding damage and non-host insects, repeatedly return to the non-host-infested plants, or oviposit in non-hosts (Vos et al. 2006; Bukovinszky et al. 2012).

Search templates are constantly updated during foraging (Aartsma et al. 2019) and foraging experience on host- and/or non-host-infested plants may help parasitoids to fine-tune their foraging behaviour, hence reducing the distraction by non-host-infested plants (De Rijk et al. 2018). *C. glomerata* readily learns volatile cues during host oviposition and already forms long-term memory 4 h after a single oviposition in *Pieris brassicae* (Smid et al. 2007), a learning strategy that is thought to be adaptive during foraging for a patchily distributed gregarious host (Hoedjes et al. 2011; Smid and Vet 2016). A positive effect of assortative learning of host-induced HIPVs was found both in the presence and absence of background vegetation (Kruidhof et al. 2015). Similarly, foraging and oviposition experience strongly increased foraging efficiency of *C. glomerata* both in the presence and absence of non-host-infested potato plants (Perfecto and Vet 2003). The study of de Bruijn et al. (2018), however, showed that learning can also result in decreased foraging efficiency of *C. glomerata* if the learned information is unreliable because non-hosts are feeding on the plant species associated with oviposition success, while the hosts are found on a different plant species. Reliable information (hosts are feeding on the learned plant species, non-hosts are feeding on a different species) was not found to increase foraging success in this study, which may be due to the training procedure in which parasitoids were not exposed to non-host-infested plants prior to the experiment. The study of Vet et al. (1998) suggests that parasitoids may need complete experience with host- and non-host-related cues to forage efficiently in complex environments if volatile blends only differ quantitatively and not qualitatively.

Here, we studied how the presence of non-host-infested plants of the same species as the host-infested plant influences parasitoid foraging behaviour in a complex environment with overlapping odour plumes to see if plants infested by non-host caterpillars confuse foraging parasitoids. We further tested if previous host oviposition and/or non-host experience increases parasitoid foraging efficiency in the presence of non-host-infested plants. To quantify confusion, we recorded if parasitoids landed on the non-host-infested plants, if they attacked non-host caterpillars and measured the time they needed to find the host-infested plant. Oviposition in non-host caterpillars, as observed by de Bruijn et al. (2018), may have quite severe fitness consequences, because these eggs will not develop and resources used to produce the eggs are wasted. An increase in foraging time, however, can also be expected to negatively influence parasitoid fitness, because adverse weather conditions were found to limit the time available for foraging (Weisser et al. 1997; Vosteen et al. 2019). We expected that non-host-infested plants would confuse the foraging parasitoids and that experienced parasitoids would spend less time during inspection of the non-host-infested plants, especially after they had gained complete experience on host- and non-host-infested plants.

## Material and methods

### Organisms

The parasitoid *C. glomerata*, caterpillars of the large cabbage white *P. brassicae* and the cabbage moth *Mamestra brassicae* were obtained from cultures maintained at the Laboratory of Entomology, Wageningen University. All insects were reared on Brussels sprouts plants (*Brassica oleracea* L. var. *gemmifera* cultivar Cyrus) under climate-controlled conditions (L16:D8 photoperiod, at 21 ± 1 °C and 50–70% relative humidity). The parasitoid culture is replaced every summer with *C. glomerata* that emerged from *P. brassicae* caterpillars that had been placed in the field site at Wageningen University, the Netherlands to avoid any long-term effects of parasitoid breeding under laboratory conditions. To obtain a new generation of parasitoids for experiments and continuation of the culture, a Brussels sprouts leaf with approximately 200 first instar *P. brassicae* was placed in the parasitoid rearing cage and parasitoids were allowed to oviposit for 5–10 min. After pupation, parasitoid cocoons were transferred to screen cages (30 × 30 × 30 cm, Bugdorm, Taiwan) in a climate cabinet at 24 ± 1 °C, LD12:D12 away from plant odours. Emerging parasitoids were supplied with water and honey. Females were used at the age of 2–5 days and were separated from males 1 day before the experiment.

Four- to five-week-old Brussels sprout plants (*B. oleracea* L. var. *gemmifera* cultivar Cyrus) were used as an odour source in the experiment. The plants were either undamaged or infested by ten 1- to 2-day-old *P. brassicae* (hosts) or 10 *M. brassicae* (non-hosts) 1 day before the experiment. In the first experiment (row experiment with inexperienced parasitoids), *M. brassicae* were 4–5 days old, but since they caused more feeding damage than *P. brassicae* in this experiment, younger (1–3 days old) *M. brassicae* were used in all subsequent experiments to achieve a similar amount of damage compared to *P. brassicae* feeding. Caterpillars were always placed on one of the newly emerging leaves and were allowed to freely disperse on the plant. While *P. brassicae* usually fed gregariously close to the release site, *M. brassicae* moved all over the plant and caused highly dispersed feeding damage. On each experimental day, a fresh set of plants was used.

### Experimental set-up

All experiments were conducted in a wind tunnel (size: 200 × 60 × 60 cm, for a detailed description see Geervliet et al. 1994) at the following abiotic conditions: 0.1 m/s wind speed, 24–26 °C, 59–64% RH. Parasitoids were released individually inside a horizontal glass cylinder (30 cm long, 15 cm diameter) with both ends open to prevent parasitoids from immediately flying towards the ceiling (Geervliet et al. 1994). The glass cylinder was located downwind from the plants.

#### Foraging efficiency in the presence of overlapping odour plumes: plant row experiments

To test how the presence of non-host-infested plants influences parasitoid foraging efficiency in a complex environment with overlapping odour plumes, we released wasps in an environment with a host-infested plant and either three undamaged or three non-host-infested plants, and recorded if the parasitoids landed on the non-host-infested plants, attacked non-host caterpillars and measured the foraging time spend until the host-infested plant was found.

**Fig. 1:**
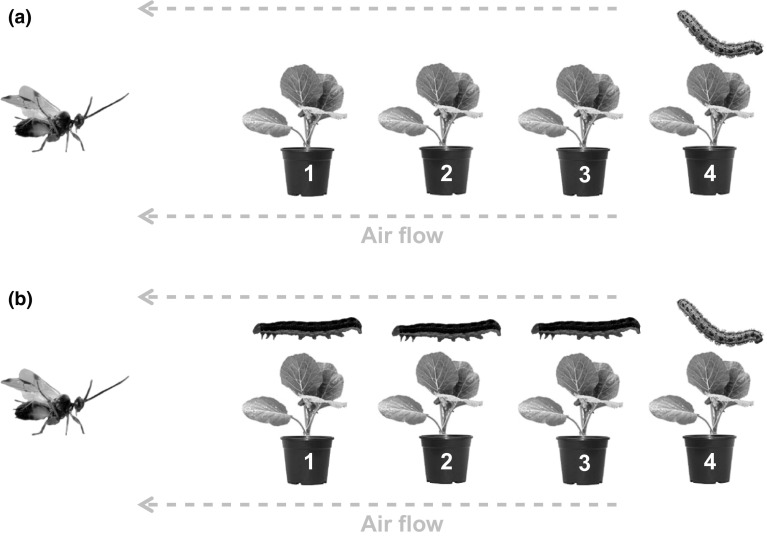
Set-up of the row experiment in the wind tunnel. The first three plants were either undamaged (**a**) or infested by the non-host caterpillar *Mamestra brassicae* (**b**), while the fourth plant contained the host caterpillars *Pieris brassicae*. *Cotesia glomerata* wasps were individually released at the downwind side of the plant row

The four plants were arranged in a row following the direction of the air flow. The first plant was located 50 cm away from the release site and the distance between the plants was 25 cm, measured from stem to stem. Plants were positioned in such a way that the leaves did not touch each other. The first three plants from the release point were either undamaged or non-host infested, while the last plant was infested with host caterpillars (Fig. 1). Female parasitoids were released from a glass vial that was placed upright in the release cylinder to allow the parasitoid to climb out. Parasitoids that had not taken flight within 6 min after they had climbed out of the glass vial and those that flew directly to the ceiling of the wind tunnel were considered as non-responsive. Those parasitoids that started foraging were observed until they oviposited into a host caterpillar or for a maximum of 30 min. First landing choice of the parasitoid and all subsequent landings until host oviposition as well as all non-host attacks were documented. Additionally, foraging time until landing on the host-infested plant was recorded. If a parasitoid rested for more than 5 min on a plant or flew to the walls or ceiling of the wind tunnel and stayed there for more than a minute, observations were terminated and the parasitoid was scored as non-successful forager.

In the first experiment, the effect of the presence of non-host-infested plants on foraging behaviour of inexperienced parasitoids was tested in comparison to undamaged plants. On five experimental days a total of 21 responding wasps were tested for both treatments. Contrary to the other experiments, each parasitoid was observed until it oviposited in a host caterpillar, even if the 30 min of maximum observation time were exceeded, to get an estimation on the absolute increase in foraging time caused by non-host-infested plants.

In the second experiment, the effect of parasitoid experience on foraging efficiency in the presence of non-host-infested plants was tested. On seven experimental days, a total of 36 oviposition experienced, 37 non-host experienced and 45 inexperienced wasps were tested, resulting in 31, 32, and 32 responding parasitoids, respectively. Parasitoids had been conditioned on the day before they were used in the experiments. For gaining oviposition experience, individual female wasps were released in a petri-dish containing a piece of leaf with 20–30 one- to two-day-old *P. brassicae* and were allowed to oviposit once (conditioning procedure adapted from Geervliet et al. (1998)). The leaf piece had been taken from a Brussel sprouts plant heavily infested with 1- to 2-day-old *P. brassicae* caterpillars just before the conditioning was started.

Parasitoids that were conditioned with non-hosts had been kept for 15 min in a glass vial with a piece of a Brussel sprouts leaf that had been excised just before the conditioning started and contained approximately 20 one- to three-day-old caterpillars of the non-host *M. brassicae*. During the non-host conditioning, the parasitoids were usually sitting motionless on the leaf or the wall of the glass and did not start to forage on the non-host-infested leaves. No non-host attacks were observed during this conditioning treatment.

The third experiment was conducted to test if complete or incomplete experience influences foraging efficiency of parasitoids in the presence of non-host-infested plants. A total of 30 wasps with complete experience, 26 wasps with incomplete experience and 46 inexperienced wasps were tested on ten experimental days, resulting in 29, 25 and 34 responding parasitoids, respectively. Since the artificial training situation in which parasitoids were exposed to caterpillars on excised leaves may not be optimal to learn host- and non-hosted-induced HIPVs, we decided to use parasitoids which had gained experience in a relatively natural training situation. Therefore, we used parasitoids which had been used as inexperienced wasps in two-choice tests with undamaged, host-infested and non-host-infested plants (see two-choice tests for the exact set-up). Only those wasps which had managed to find the host-infested plant and oviposited were considered as experienced and the unsuccessful individuals were discarded. Those parasitoids that had been foraging in the host-infested vs. undamaged situation were considered to have incomplete experience on the host-infested plant alone, while those previously tested in the host-infested vs. non-host-infested situation were considered to have complete experience if they had landed both on the host- and on the non-host-infested plants and oviposited in the host caterpillars. By discarding those parasitoids that did not land on the non-host-infested plant (37 out of 79 parasitoids) or failed to oviposit in the host caterpillars (host- vs. non-host-infested plants (complete experience): 13 out of 79; host-infested vs. undamaged plants (incomplete experience): 5 out of 51), we may have introduced an unintended bias: parasitoids that passed this training procedure may be a non-random subsample of the cultured parasitoids. Such a bias, however, is unavoidable in a relatively natural training situation that allows for non-responsiveness and unsuccessful foraging.

#### Foraging efficiency in two-choice situations

Two-choice assays were conducted to test how experience influences the host-plant preferences of parasitoids in a simple foraging environment. Two plants were placed at the sides of the wind tunnel, 1 m away from the downwind release point. One plant was always infested by host caterpillars, while the second plant was either non-host infested or undamaged, which resulted in two different choice situations (host infested vs. undamaged and host vs. non-host infested). Plant location was switched after every three replications to account for a potential bias in side preference by parasitoids. Behavioural observations were conducted as described in the row experiment.

The effect of oviposition experience was tested in the first two-choice assay. On six experimental days, a total number of 43 oviposition-experienced and 53 inexperienced parasitoids were tested in the host-infested vs. non-host-infested foraging situation, resulting in 28 and 38 responding parasitoids, respectively. In the host vs. non-damaged situation, 51 inexperienced parasitoids were tested, resulting in 32 responding parasitoids.

In the second two-choice assay, the effect of non-host experience was tested during six experimental days. A total number of 46 oviposition-experienced and 61 inexperienced parasitoids were tested in the host-infested vs. non-host-infested foraging situation, resulting in 35 and 41 responding parasitoids, respectively. In the host vs. non-damaged situation, 37 inexperienced parasitoids were tested, resulting in 22 responding parasitoids.

### Statistical analysis

All data were analysed with R version 3.3.1 R (R Development Core Team 2014).

To account for variation between the different experimental days, all data were analysed with generalised linear mixed-effect models (glmm) or linear mixed-effect models (lme) with day as a random factor (random intercept). *p* values for explanatory variables of glmms were obtained by sequentially deleting explanatory variables and comparison of the more complex model with the simpler model (Zuur et al. 2009). In cases of significant differences, factor level reductions were used to reveal differences between levels of a treatment (Crawley 2013).

Parasitoid responsiveness, foraging success and the occurrence of a specific behaviour (first landing on host-infested plant in two-choice assays, direct flights to the host-infested plant in the row experiment, non-host attacks) were treated as presence/absence of data and were analysed with glmms with a binomial error distribution [glmer function of the lme4 package; Bates et al. (2014)]. Parasitoid experience was used as fixed effect in the row experiments, while in the two-choice assays, the fixed effect was a combination of parasitoid experience and foraging situation (host infested vs. non-host infested or host-infested vs. undamaged).

To see if parasitoids had a preference for one of the offered plants, a glm with a binomial error structure was used to compare the observed choices with an artificial dataset where each plant was chosen in 50% of the cases.

Foraging time was log-transformed to achieve homogeneity of variances and was analysed with linear mixed-effect models (lme function of the nlme package; Pinheiro et al. 2013). Parasitoid experience and foraging situation were used as fixed effect in the analysis of the two-choice assay. For the different row experiments, different fixed factors were used to analyse foraging time. We included the fixed effect of the presence/absence of non-host-infested plants to analyse the effect of non-hosts in the first setup. For the analysis of the row experiments on learning, we included the fixed effects parasitoid experience and occurrence of direct flights to the host-infested plant.

## Results

### Foraging efficiency in complex environments: influence of non-host-infested plants

**Fig. 2:**
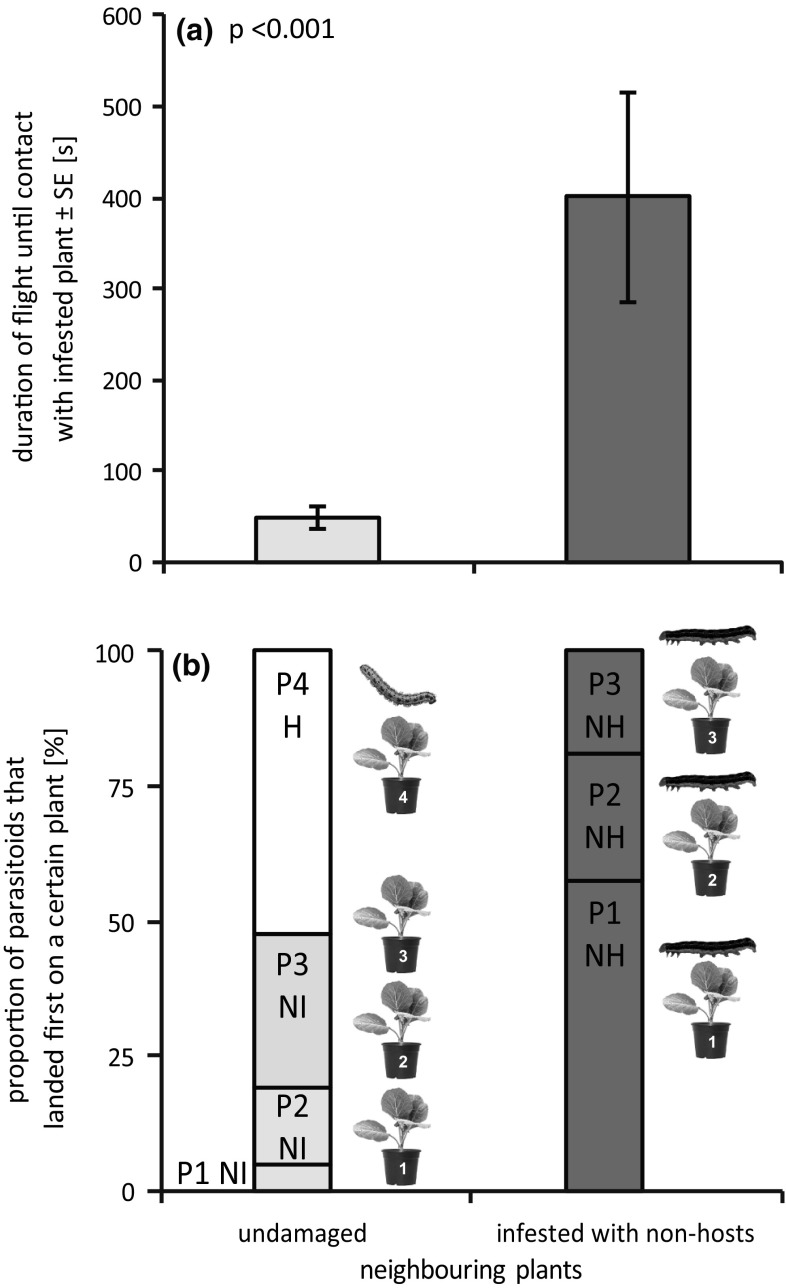
Influence of non-host-infested plants on **a** duration of *Cotesia glomerata* foraging until locating the host-infested plant and on **b** the first landing choice of the parasitoid. P1-4: plant 1–4, H: host infested, NI: non-infested, NH: non-host infested

Foraging time increased by eightfold if parasitoids had to pass three non-host-infested plants to reach the host-infested target plant compared to a situation where undamaged plants were placed in between the release point and the host-infested plant (lme: *F* = 51.904; *p* < 0.001, Fig. 2a).

While 50% of parasitoids passed the undamaged plants and directly flew to the host-infested plant, no parasitoids performed direct flights in the presence of non-host-infested plants. Instead, parasitoids frequently landed on one or several of the non-host-infested plants before reaching the host-infested target plant (Fig. 2b).

### Foraging efficiency in complex environments: influence of experience

To test if foraging experience influences parasitoid foraging efficiency in the presence of non-host-infested plants, parasitoids with oviposition experience and non-host experience were tested in the experimental set-up where they had to pass non-host-infested plants before they would reach the host-infested target plant.

**Fig. 3:**
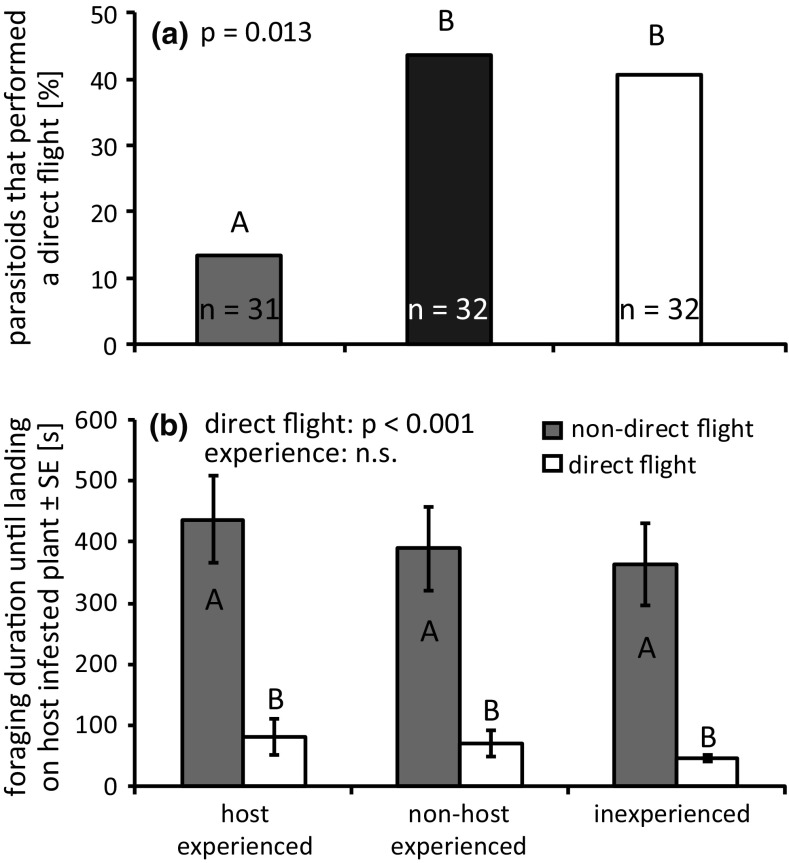
Influence of experience on **a** the proportion of *Cotesia glomerata* that performed direct flights to the host-infested target plant and **b** on foraging duration until landing on the host-infested plant. Different letters indicate significant differences. *n* number of responding parasitoids

80% of the tested parasitoids started foraging in this experiment and this proportion was not influenced by experience (binomial glmm: *χ*^2^ = 3.11; *p* = 0.211). Contrary to the previous experiment, some parasitoids flew directly to the host-infested plant and did not land on any of the non-host-infested plants. Parasitoids with oviposition experience performed fewer direct flights than inexperienced and non-host-experienced wasps (binomial glmm: *χ*^2^ = 8.66; *p* = 0.013, Fig. 3a).

Foraging time until landing on the host-infested plant differed between direct and non-direct flights (lme: *F* = 94.99; *p* < 0.001, Fig. 3b), but was not influenced by parasitoid experience (lme: *F* = 1.03; *p* = 0.361). Regardless of experience (binomial glmm: *χ*^2^ = 3.11; *p* = 0.212), 90% of foraging parasitoids successfully located and attacked host caterpillars. When making indirect flights, parasitoids occasionally attacked non-host caterpillars (inexperienced parasitoids: 1, non-host experienced: 1, oviposition experienced: 4), but the effect of experience was weak and not statistically significant (binomial glmm: *χ*^2^ = 3.5896; *p* = 0.166).

### Foraging efficiency in complex environments: effect of training situation and completeness of information

To test whether parasitoid host location would improve by completeness of information about HIPVs in the environment, we used parasitoids that had gained foraging experience in preceding two-choice experiments in the wind tunnel and tested them in the row setup with non-host-infested plants placed in between the release point and the host-infested plant.

Contrary to the previous experiments with parasitoids that were trained on infested leaves, experience had an effect on the proportion of responding parasitoids in this experiment (binomial glmm: *χ*^2^ = 10.86; *p* = 0.004). 74% of inexperienced parasitoids and 96% of parasitoids with complete (host and non-host) or host experience started foraging.

**Fig. 4:**
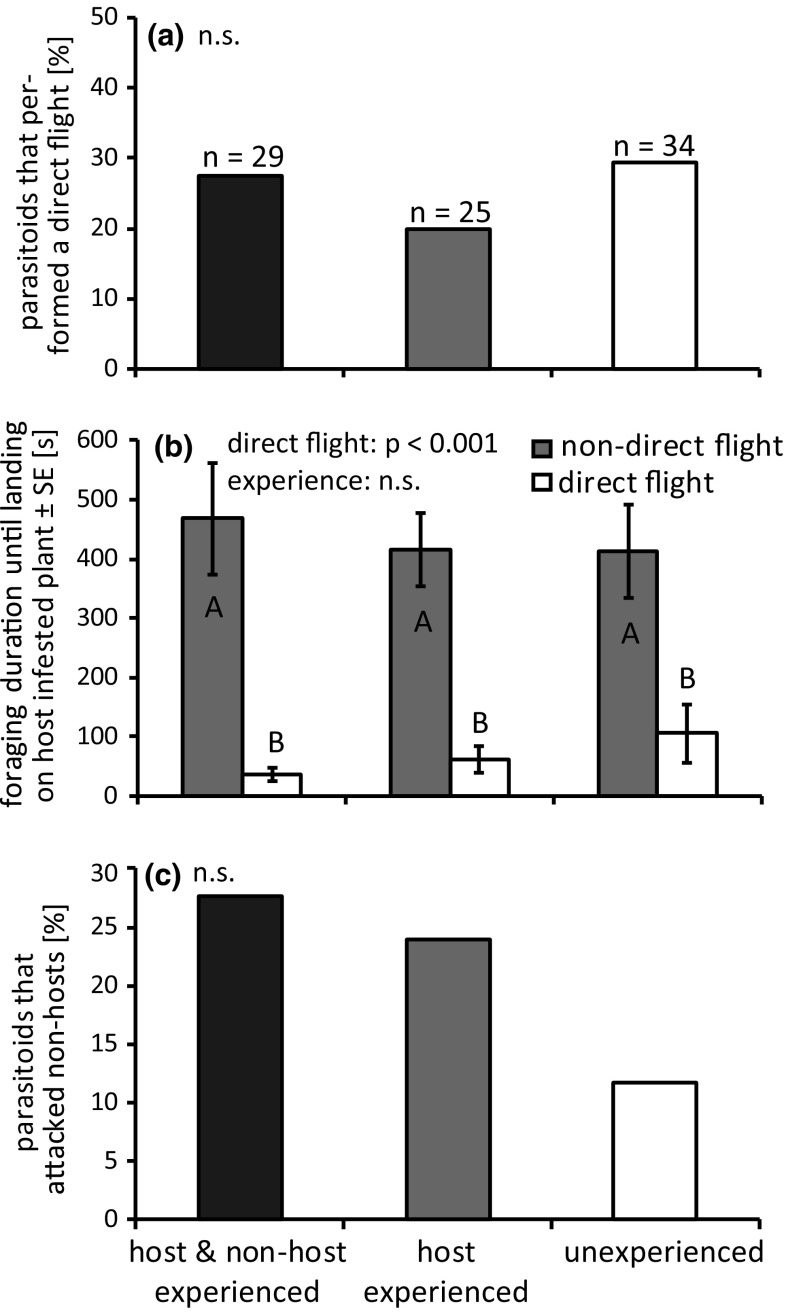
Influence of foraging experience gained in the wind tunnel on **a** the proportion of *Cotesia glomerata* that perform direct flights to the host-infested target plant, **b** on foraging duration until landing on the host-infested plant and **c** on the proportion of parasitoids that attacked non-host caterpillars. Different letters indicate significant differences. *n* number of responding parasitoids

More than twice as many experienced than inexperienced parasitoids attacked non-host caterpillars, but this difference was not significant (binomial glmm: *χ*^2^ = 2.82; *p* = 0.244, Fig. 4c). Foraging experience gained during two-choice assays also did not influence the number of direct flights to the host-infested target plant (binomial glmm: *χ*^2^ = 0.86; *p* = 0.650, Fig. 4a). Foraging time until landing on the target plant differed between direct and non-direct flights (lme: *F* = 57.16; *p* < 0.001, Fig. 4b), but was not influenced by parasitoid experience (lme: *F* = 0.66; *p* = 0.719). 89% of foraging parasitoids successfully located and attacked host caterpillars. This proportion was not influenced by experience (binomial glmm: *χ*^2^ = 1.52; *p* = 0.467).

### Foraging efficiency in two-choice situations: influence of oviposition and non-host experience

In two-choice wind tunnel experiments, experience did not influence the proportion of responding parasitoids, neither in the experiment with oviposition-experienced nor in the experiment with non-host-experienced parasitoids (binomial glmm–oviposition experience: *χ*^2^ = 2.096; *p* = 0.351, non-host experience: *χ*^2^ = 0.149; *p* = 0.475). 68% and 66% of parasitoids started foraging in these experiments, respectively.

**Fig. 5:**
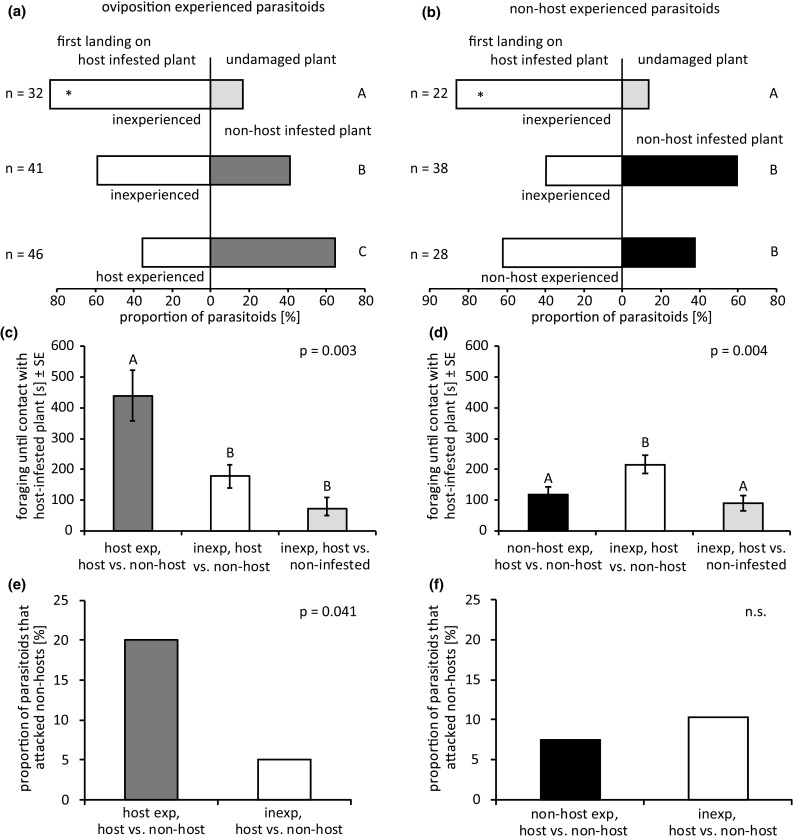
Influence of foraging situation and oviposition experience (**a**, **c**, **e**) or non-host experience (**b**, **d**, **f**) on foraging behaviour of *Cotesia glomerata* in two-choice assays: **a**, **b** landing preference for host-infested, non-host-infested and non-infested plants, **c**, **d** foraging duration, **e**, **f** proportion of non-host attacks. Different letters indicate significant differences between treatments. Asterisk indicates a significant difference of the observed landing preference to a hypothetical situation where parasitoids do not distinguish between the two plants (50% of parasitoids land first on the host-infested plant). *n* indicates the number of responding parasitoids that were observed during the experiment

Inexperienced parasitoids significantly preferred the host-infested over the undamaged plant (binomial glm: LRT = 7.76, *p* = 0.005), while neither inexperienced nor oviposition-experienced parasitoids preferred the host-infested to the non-host-infested plant (binomial glm–oviposition experience: LRT = 1.51, *p* = 0.219, inexperienced: LRT = 0.44, *p* = 0.505). The proportion of parasitoids that landed first on the host-infested plants differed between the three two-choice assays (binomial glmm: *χ*^2^ = 16.501; *p* < 0.001, Fig. 5a) and was highest (83%) when inexperienced parasitoids were allowed to choose between host-infested and undamaged plants. The proportion of parasitoids that chose the host-infested over the non-host-infested plant decreased significantly after oviposition experienced, from 59 to 35%.

Oviposition-experienced parasitoids took much longer to locate the host-infested plant than inexperienced parasitoids (lme: *F* = 6.228; *p* = 0.003, Fig. 5c), which were equally efficient in the presence of an undamaged or a non-host-infested plant. During foraging, parasitoids occasionally attacked non-host caterpillars, which happened significantly more often if parasitoids had oviposition experience (binomial glmm: *χ*^2^ = 4.178; *p* = 0.041, Fig. 5e).

Similar to the previous two-choice experiment, inexperienced parasitoids significantly preferred the host-infested over the undamaged plant (binomial glm: LRT = 5.68, *p* = 0.017), while neither inexperienced nor oviposition-experienced parasitoids preferred the host-infested to the non-host-infested plant (binomial glm–non-host experience: LRT = 0.63, *p* = 0.426, inexperienced: LRT = 0.48, *p* = 0.490). The proportion of parasitoids that landed first on the host-infested plants differed also in this experiment between the three two-choice assays (binomial glmm: *χ*^2^ = 11.62; *p* = 0.003, Fig. 5b) and was highest (86%) when inexperienced parasitoids were allowed to choose between host-infested and undamaged plants. The proportion of parasitoids that chose the host-infested over the non-host-infested plant did not differ between inexperienced (40%) and non-host-experienced parasitoids (62%).

Foraging time until location of the host-infested plant differed between the three two-choice situations (lme: *F* = 5.89; *p* = 0.004, Fig. 5d). Inexperienced parasitoids needed more time to locate the host-infested plant in the presence of a non-host-infested plant than in the presence of undamaged plant. In the presence of a non-host-infested plant, non-host-experienced parasitoids needed less time until landing on the host-infested plant than inexperienced parasitoids and they were as fast as the inexperienced parasitoids in the two-choice assay with the undamaged plant. There was no effect of experience on the proportion of parasitoids that attacked non-hosts (binomial glmm: *χ*^2^ = 0.07; *p* = 0.790, Fig. 5e).

## Discussion

In this study, we show that non-host (*M. brassicae*)-infested plants placed in-between the parasitoid and the host-containing plant confuse foraging *C. glomerata*, which results in an eightfold increase in foraging time until landing on the host-infested plant. Contrary to our expectations, neither previous oviposition experience nor non-host experience increased foraging efficiency in the presence of non-host-infested plants. Oviposition-experienced parasitoids were actually more strongly attracted towards non-host-infested plants which resulted in an increase in foraging time compared to inexperienced parasitoids.

If insects employ search templates that are so broad that they match also with cues provided by non-resources, this may result in confusion by the presence of non-resources. The two-choice assays conducted in this study show that inexperienced *C. glomerata* do not prefer HIPVs emitted from plants infested with their favourite host *P. brassicae* over HIPVs from plants infested with the non-host *M. brassicae*, thereby indicating that both HIPV blends are similar in their attractiveness. Thus, it is not surprising that inexperienced parasitoids spend quite some time to inspect the non-host-infested plants, which results in prolonged foraging until the host-infested plant is found. Confusion by non-host-infested plants appeared to be stronger in the first row experiment compared to the other row experiments. This is likely due to differences in the amount of non-host feeding damage, since the *M. brassicae* used in the first row experiment were slightly older than caterpillars in the other experiments and caused more feeding damage, which likely triggered stronger HIPV emission. This stronger HIPV emission, however, did not trigger non-hosts attacks, probably because of the larger size of the caterpillars in this experiment.

Non-suitable resources that provide cues which match with the search template of a foraging animal have a strong potential to cause confusion; yet, the effect of confusion on foraging efficiency has rarely been studied. The study of Garcia and Lemus (2012) indicates that confusion due to cue similarity may cause reduced food intake and weight loss in fish and it was repeatedly shown that parasitoids attack fewer hosts when they are foraging in an environment that contains host- and non-host-infested plants (Meisner et al. 2007; Bukovinszky et al. 2012; De Rijk et al. 2016a). Confusion caused by non-host-infested plants may be responsible for the 50% reduction in parasitization rate by *C. glomerata* observed in the presence of non-host-infested plants (Bukovinszky et al. 2012). Similarly, an increase of foraging time by 50% was even observed in a diverse plant community in which non-hosts were feeding on a plant species which are not suitable as food plant for hosts of *C. glomerata* (Perfecto and Vet 2003).

*Pieris brassicae*, the preferred host of *C. glomerata,* can be found on a number of brassicaceous host plants. Inexperienced *C. glomerata* may thus have relatively broad search template to recognise their hosts on these different plant species, which may be responsible for their inability to distinguish between HIPVs induced by the feeding of host (*P. brassicae, P. rapae*) and non-host (*M. brassicae, Plutella xylostella*) caterpillars (Geervliet et al. 1996; Shiojiri et al. 2000; Vos et al. 2001; Bukovinszky et al. 2012, this study). A recent meta-analysis showed that 38% of all tested parasitoid species respond to volatiles emitted by artificially damaged or non-host-attacked plants (Oudenhove et al. 2017). The ability of parasitoids to distinguish between HIPVs from host- and non-host-infested plants likely depends on the relative difference between the volatile blends. The composition of HIPVs emitted by herbivore damaged *Brassica rapa* predicted the diet breadth, feeding mode, and coexistence history of the herbivore damaging the plant with an accuracy of 80% or higher (Danner et al. 2018). This suggests that it may be relatively easy for parasitoids to distinguish between different types of herbivores, but difficult to discriminate between herbivores such as *P. brassicae* and *M. brassicae* that do not differ much in traits associated with feeding. It was indeed found that *C. glomerata* (Desurmont et al. 2015), *Cotesia rubecula* (Geervliet et al. 1996; Van Poecke et al. 2003), *Cotesia kariyai* (Thanikkul et al. 2017), *Microplitis mediator* (Carrasco et al. 2017; Desurmont et al. 2017) and *Aphidius ervi* (Du et al. 1996) can distinguish between host and non-host HIPV blends if the herbivores differ in at least one of the traits mentioned above. *Cotesia flavipes,* however, was not able to distinguish between HIPVs induced by its stem-boring host and a chewing herbivore (Peñaflor et al. 2017), while *Cotesia plutella* and *Cardiochiles nigriceps* preferred host-induced HIPVs over non-host-induced volatiles, despite strong similarities in herbivore traits (De Moraes et al. 1998; Peñaflor et al. 2017).

Associative learning may refine the search template of a foraging parasitoid and may allow parasitoids to deal with environments in which host- and non-host-infested plants emit relatively similar HIPVs. Contrary to our expectations, previous experience with host-infested leaves did not enable *C. glomerata* to distinguish between host- and non-host-infested plants. Instead, oviposition experience increased attraction towards non-host-infested plants and resulted in stronger confusion, indicating that oviposition experience caused a general sensitization towards HIPVs, a phenomenon that was also observed in other parasitoid studies (Geervliet et al. 1998; Peñaflor et al. 2017). Stimulus generalisation broadens the search template and enables foraging animals to cope with possible distortions of the original signal due to noise, extrinsic or intrinsic environmental interferences and allows association between similar cues (Giurfa and Menzel 2013). A general sensitization towards HIPVs after oviposition experience may ascertain that foraging parasitoids do not miss hosts that are feeding on other plant individuals that release slightly different HIPVs. Such generalised responses may come with the cost of more frequent visits to non-host-induced plants and result in reduced foraging efficiency in the presence of non-host-infested plants as found in our study. Similarly, oviposition experience decreased the foraging efficiency of *C. glomerata* when this information was non-reliable because non-hosts were feeding on the plant species associated with previous oviposition success, while hosts were feeding on a different plant species (de Bruijn et al. 2018).

To distinguish between host- and non-host-induced HIPVs, parasitoids may need complete information, for which a rewarding and a non-rewarding experience is required before parasitoids learn to distinguish minor differences in volatile blends induced by host and non-host herbivores (Vet et al. 1998). If odours differ more distinctly, a rewarding experience was found to be sufficient to enable differentiation between the two blends (Vet et al. 1998). Foraging efficiency of *C. glomerata* in the presence non-brassicaceous plants infested with non-host caterpillars indeed increased after oviposition experience (Perfecto and Vet 2003), while reliable information (hosts were feeding on the same plant species as during the training procedure) did not increase foraging efficiency if hosts and non-hosts were feeding on different brassicaceous plants (de Bruijn et al. 2018). In our experiment, responsiveness of complete- and incomplete-experienced parasitoids was significantly increased compared to inexperienced parasitoids, but none of the other tested parameters was significantly influenced by parasitoid experience. The proportion of parasitoids that performed direct flights to the host-infested plant was relatively low in this experiment, which may have prevented the detection of an experience effect. However, parasitoids with either complete or incomplete experience attacked non-hosts twice as often as inexperienced parasitoids. Thus, even with complete information, *C. glomerata* became less selective in respect to the identity of the attacked caterpillars after oviposition experience.

Another mechanism that can help foraging insects to adjust their search template according to the actual environmental conditions is to learn the association of unrewarding stimuli with the absence of rewards (non-reward learning) (Papini 2003; Kandori and Yamaki 2012). Changes of innate preferences after non-reward learning have been found in pollinators and a predatory bug (Kandori and Yamaki 2012 and cited references; Ardanuy et al. 2016), but until now have not been documented in parasitoids (Wardle and Borden 1989; Costa et al. 2010; Desurmont et al. 2017). A 15-min exposure to a non-host-infested leaf resulted in a slightly decreased preference of *C. glomerata* for non-host-infested plants in our two-choice assay, but did not increase foraging efficiency in the more complex foraging situation where parasitoids had to pass the non-host-infested plants before they reached the host-infested plant. A decreased response towards non-rewarding stimuli can be expected to be most beneficial in environments where non-rewarding stimuli are abundant and repeated non-rewarding experience was shown to increase the avoidance of non-rewarding stimuli in pollinators (Kandori and Yamaki 2012). Similarly, repeated encounters with non-host-infested plants may further decrease the attractiveness of non-host-infested plants, thereby increasing the foraging efficiency of parasitoids in complex environments where non-host-infested plants dominate. *C. glomerata* typically finds its preferred host *P. brassicae* that is gregarious and patchily distributed in such a non-host-dominated environment (Le Masurier 1994). In the field, only a small fraction of potential host plants was found to contain *P. brassicae* caterpillars, while nearly all of the plants were infested by other non-host herbivores including *M. brassicae* and the less preferred host *P. rapae* (Vos et al. 2001). This also means that *C. glomerata* finds its host frequently on plants co-infested by other herbivores that may affect the plant volatile blend. In two-choice assays *C. glomerata* often preferred dual infested plants that contained host plus non-host herbivores over non-host-infested plants (Shiojiri et al. 2000; Bukovinszky et al. 2012; De Rijk et al. 2016a, b, c). Similarly, foraging efficiency was not influenced by non-host-infested plants if the hosts were feeding on a dual infested plant (De Rijk et al. 2016b, c; but see Bukovinszky et al. 2012). These studies suggest that *C. glomerata* is adapted to forage in the presence of non-host-infested plants if the hosts feed together with non-hosts on the same plant. We speculate that stimulus generalisation after a positive reward combined with learning to discriminate HIPVs induced by non-hosts after non-rewarding experience allow *C. glomerata* to maximise host location success in these complex environments.

This study shows that attractive HIPVs emitted by non-host-infested plants have a strong potential to confuse foraging parasitoids and that rewarding experience may result in general sensitization towards HIPVs, thereby increasing confusion by non-host-infested plants, while non-rewarding experience resulted in a slightly reduced attraction towards non-host-related cues. It can be expected that the effect of confusing information is context dependent and a next step could be to test if the confusing effect of non-host-infested plants depends on the spatial scale. Attractive HIPVs from non-host-infested plants that result in confusion during within patch foraging may increase the long-range attraction towards the respective plant patch and thus, may make it more likely to find a host-containing patch.
